# Mass spectrometry imaging of L-[ring-^13^C_6_]-labeled phenylalanine and tyrosine kinetics in non-small cell lung carcinoma

**DOI:** 10.1186/s40170-021-00262-9

**Published:** 2021-06-11

**Authors:** Jianhua Cao, Benjamin Balluff, Martijn Arts, Ludwig J. Dubois, Luc J. C. van Loon, Tilman M. Hackeng, Hans M. H. van Eijk, Gert Eijkel, Lara R. Heij, Zita Soons, Steven W. M. Olde Damink, Ron M. A. Heeren

**Affiliations:** 1 Maastricht MultiModal Molecular Imaging institute (M4I), Maastricht University, Universiteitssingel 50, 6229 ER Maastricht, The Netherlands; 2 Department of General Surgery (NUTRIM), Maastricht University, Maastricht, The Netherlands; 3 The M-Lab, Department of Precision Medicine (GROW), Maastricht University, Maastricht, The Netherlands; 4 Department of Human Biology (NUTRIM), Maastricht University, Maastricht, The Netherlands; 5 Department of Biochemistry (CARIM), Maastricht University, Maastricht, The Netherlands; 6 Department of General, Gastrointestinal, Hepatobiliary and Transplant Surgery, RWTH Aachen University Hospital, Aachen, Germany; 7 Institute of Pathology, University Hospital RWTH Aachen, Aachen, Germany; 8 Joint Research Center for Computational Biomedicine , RWTH Aachen University Hospital , Aachen, Germany

**Keywords:** L-[ring-^13^C_6_]-Phenylalanine, L-[ring-^13^C_6_]-Tyrosine, Amino acids, Isotope labeling, Tumor, Mass spectrometry imaging

## Abstract

**Background:**

Metabolic reprogramming is a common phenomenon in tumorigenesis and tumor progression. Amino acids are important mediators in cancer metabolism, and their kinetics in tumor tissue are far from being understood completely. Mass spectrometry imaging is capable to spatiotemporally trace important endogenous metabolites in biological tissue specimens. In this research, we studied L-[ring-^13^C_6_]-labeled phenylalanine and tyrosine kinetics in a human non-small cell lung carcinoma (NSCLC) xenografted mouse model using matrix-assisted laser desorption/ionization Fourier-transform ion cyclotron resonance mass spectrometry imaging (MALDI-FTICR-MSI).

**Methods:**

We investigated the L-[ring-^13^C_6_]-Phenylalanine (^13^C_6_-Phe) and L-[ring-^13^C_6_]-Tyrosine (^13^C_6_-Tyr) kinetics at 10 min (*n* = 4), 30 min (*n* = 3), and 60 min (*n* = 4) after tracer injection and sham-treated group (*n* = 3) at 10 min in mouse-xenograft lung tumor tissues by MALDI-FTICR-MSI.

**Results:**

The dynamic changes in the spatial distributions of 19 out of 20 standard amino acids are observed in the tumor tissue. The highest abundance of ^13^C_6_-Phe was detected in tumor tissue at 10 min after tracer injection and decreased progressively over time. The overall enrichment of ^13^C_6_-Tyr showed a delayed temporal trend compared to ^13^C_6_-Phe in tumor caused by the Phe-to-Tyr conversion process. Specifically, ^13^C_6_-Phe and ^13^C_6_-Tyr showed higher abundances in viable tumor regions compared to non-viable regions.

**Conclusions:**

We demonstrated the spatiotemporal intra-tumoral distribution of the essential aromatic amino acid ^13^C_6_-Phe and its de-novo synthesized metabolite ^13^C_6_-Tyr by MALDI-FTICR-MSI. Our results explore for the first time local phenylalanine metabolism in the context of cancer tissue morphology. This opens a new way to understand amino acid metabolism within the tumor and its microenvironment.

**Supplementary Information:**

The online version contains supplementary material available at 10.1186/s40170-021-00262-9.

## Introduction

Cancer cells are known to exhibit unusual metabolic activity to sustain their proliferation [1]. A well-known example is the non-essential amino acid glutamine, which is associated with neoplastic proliferation [2]. It is also known that phenylalanine consumption correlates with the growth of tumor cell lines and negative patient outcomes [3–12]. Plasma phenylalanine concentrations are elevated in patients with cancer [12]. Phenylalanine is an indispensable amino acid and phenylalanine flux in the body is entirely derived from the diet and cellular protein turnover. It is also needed to synthesize the amino acid tyrosine (Tyr) by the phenylalanine hydroxylase enzyme mainly in the liver and kidney [13]. Isotopically labeled phenylalanine has been used in tracer studies as a measure of protein synthesis [14, 15] and liver function [16, 17]. Phenylalanine metabolism, however, in relation to the morphology of cancer tissue has not been explored yet.

The molecular and cellular heterogeneity in a tumor plays a crucial role in cancer treatment efficacy and outcome [18, 19]. Current strategies to personalize treatment response use genomics data [20] which is not able to reflect the dynamics of metabolic processes in the context of the spatial intratumor heterogeneity. Mass spectrometry imaging (MSI) enables the in situ visualization of metabolites in biological tissue specimens. Adding isotopically labeled versions of target compounds allows the quantitative study of spatiotemporal metabolic dynamics in these tissues [17]. Multi-isotope imaging mass spectrometry (MIMS) has been applied to study the heterogeneity of glucose and glutamine utilization in murine tumors recently [21]. However, matrix-assisted laser desorption/ionization (MALDI), a softer ionization technique without suffering from extensive fragmentation and complexity of interpretation of mass spectra, is increasingly applied in biomedical research [22]. We have recently used this method to track the hepatocellular incorporation of L-[ring-^13^C_6_]-Phenylalanine (^13^C_6_-Phe) and its metabolite L-[ring-^13^C_6_]-Tyrosine (^13^C_6_-Tyr) [17].

In this study, we used our previously developed MALDI-MSI method of ^13^C_6_-Phe [17] to construct spatial and dynamic metabolic flux maps in relation with spatial tumor heterogeneity in a human non-small cell lung carcinoma xenograft model.

## Materials and methods

### Animal experiments

A total of 14 adult female immune-compromised Crl:NU-Foxn1^nu^ nu/nu nude mice (Charles River, Den Bosch, The Netherlands) were used. Human NCI-H460 non-small cell lung carcinoma (NSCLC) cells suspended in matrigel (BD Biosciences, Breda, The Netherlands) were injected subcutaneously into the flank region of each mouse. Tumor volume was monitored 3 times per week using a Vernier caliper. All animals had unrestricted access to food and water before injection. They received a regular chow diet, which contains 12% fat, 27% protein, and 61% carbohydrate based on calories. Eleven mice were injected with ^13^C_6_-Phe (Cambridge Isotope Laboratories, Andover, MA, USA) at a dose of 1.0 micromole/g body weight, and 3 additional mice were injected with normal saline into the lateral tail vein when the tumors reached 1000 mm^3^. The tracer-infused mice were subsequently sacrificed at 10 min (*n* = 4), 30 min (*n* = 3), and 60 min (*n* = 4) after injection, and sham-treated mice (*n* = 3) were sacrificed after 10 min. Tumors were rapidly dissected, snap-frozen with liquid nitrogen, and stored at − 80 °C until cryo-sectioning. All experimental procedures were approved by the Animal Ethical Committee of the Maastricht University.

### Tissue sectioning

Tumor tissues were sectioned at 10 μm using a cryotome (Leica, Rijswijk, The Netherlands) at − 20 °C, thaw-mounted onto indium−tin oxide coated glass slides (CG-40IN-S115, Delta Technologies, Loveland, CO, USA), and stored at − 80 °C until further measurement.

### On-tissue derivatization and matrix application

P-N,N,N-trimethylamonioanilyl N-hydroxysuccinimidylcarbamate iodide (TAHS) on-tissue derivatization was applied to tissue sections prior to matrix application as described by Arts et al. [17] with slight modifications: fresh frozen tissue sections were dried in a vacuum desiccator for 15 min. Subsequently, a TAHS solution of 1.25 mg/mL in acetonitrile was sprayed onto the sections using an automated, temperature-controlled spraying system (TM-sprayer, HTX Technologies, Chapel Hill, NC, USA). Six layers were sprayed at 55 °C with a constant flow rate of 0.1 mL/min and at a speed of 1200 mm/min. Next, all tumor sections were incubated at 55 °C in a humid environment (methanol to water = 1:1, v/v) for 24 h.

A 30 mg/mL 2,5-dihydroxybenzoic acid (DHB, Sigma-Aldrich, St. Louis, MO, USA) matrix solution in methanol/water (7:3, v/v) containing 0.2% trifluoroacetic acid was applied in six layers with the HTX sprayer at 85 °C with a fixed flow rate of 0.1 mL/min, followed by immediate mass spectrometry imaging measurements.

### Mass spectrometry imaging experiments

High mass resolution (R = 1.5E5 at *m/z* 200) matrix-assisted laser desorption/ionization Fourier-transform ion cyclotron resonance mass spectrometry imaging (MALDI-FTICR-MSI) experiments were performed with a Solarix 9.4 T (Bruker Daltonics, Bremen, Germany). MSI data were acquired within a mass range of *m/z* 100–1200 (1E6 data points) in positive ionization mode and in magnitude mode with a 75-μm spatial raster width. The laser operated at a laser power of 18% and a frequency of 2000 Hz with 50 shots accumulated per pixel. Data acquisition was controlled using ftmsControl and FlexImaging 4.1 (Bruker Daltonik, Bremen, Germany).

### LC-MS and GC-C-IRMS measurements

The enrichment of free amino acids (^13^C_6_-Phe and ^13^C_6_-Tyr) and protein-bound ^13^C_6_-Phe were measured in tumor tissue homogenates with liquid chromatography–mass spectrometry (LC-MS) [23] and gas chromatography combustion isotope ratio mass spectrometry (GC-C-IRMS) [24] using the same protocols as described by Van et al. and Arts et al., respectively to complement and validate the MSI data.

### Histological staining

After MSI measurement, all tissue sections were washed with 70% ethanol for 30 s to remove the matrix prior to hematoxylin and eosin staining (H&E). The samples were rehydrated in MilliQ water, followed by 3 min in hematoxylin (Merck, Darmstadt, Germany), 1 min in distilled water, and 30 s in eosin (Merck, Darmstadt, Germany). Then, all sections were dehydrated in a graded ethanol series and followed by clearance for 2 min in xylene. Coverslips were mounted onto the slides with Entellan mounting medium (Merck, Darmstadt, Germany), and all sections were air-dried overnight at room temperature. The H&E stained slides were scanned using a digital slide scanner (Mirax Desk, Zeiss, Jena, Germany) and a pathologist annotated viable tumor and non-viable tumor regions digitally in the scanned images. Next, the digitalized H&E images were manually co-registered to the MSI data using FlexImaging 4.1 (Bruker Daltonics, Bremen, Germany).

### Data analysis

The tracer-to-tracee ratio (TTR) and the molar percentage excess (MPE) values of ^13^C_6_-Phe and ^13^C_6_-Tyr, and ratios of MPE (Tyr) to MPE (Phe) were calculated for every pixel individually using a custom MATLAB script (MATLAB R2014b, Mathworks, Natick, MA, USA) as described by Arts et al. [17]. This resulted in tabular ASCII files that can be imported for heatmap reconstructions in FlexImaging 4.1 (Bruker Daltonics, Bremen, Germany).

In parallel, all MSI data, their co-registered H&E images combined with the tumor annotations, were imported to SCiLS Lab 2020a (Bruker Daltonics). There, the peak interval width was set to 5 mDa and each pixel was normalized to its root mean square value. The average intensity (“maximum mean value”) data of annotated regions were exported.

### Metabolite identification

The human metabolome database (www.hmdb.ca) was used for assignment of identities to *m/z* values with a maximum mass tolerance of 2 ppm. Underivatized molecules were identified assuming single protonation (M = *m/z* - H^+^), while derivatized molecules ([M + TAHS]^+^) were identified based on subtracting the monoisotopic mass value of TAHS to obtain the neutral molecular weight (M = *m/z* - 177.1022394).

## Results

**Fig. 1:**
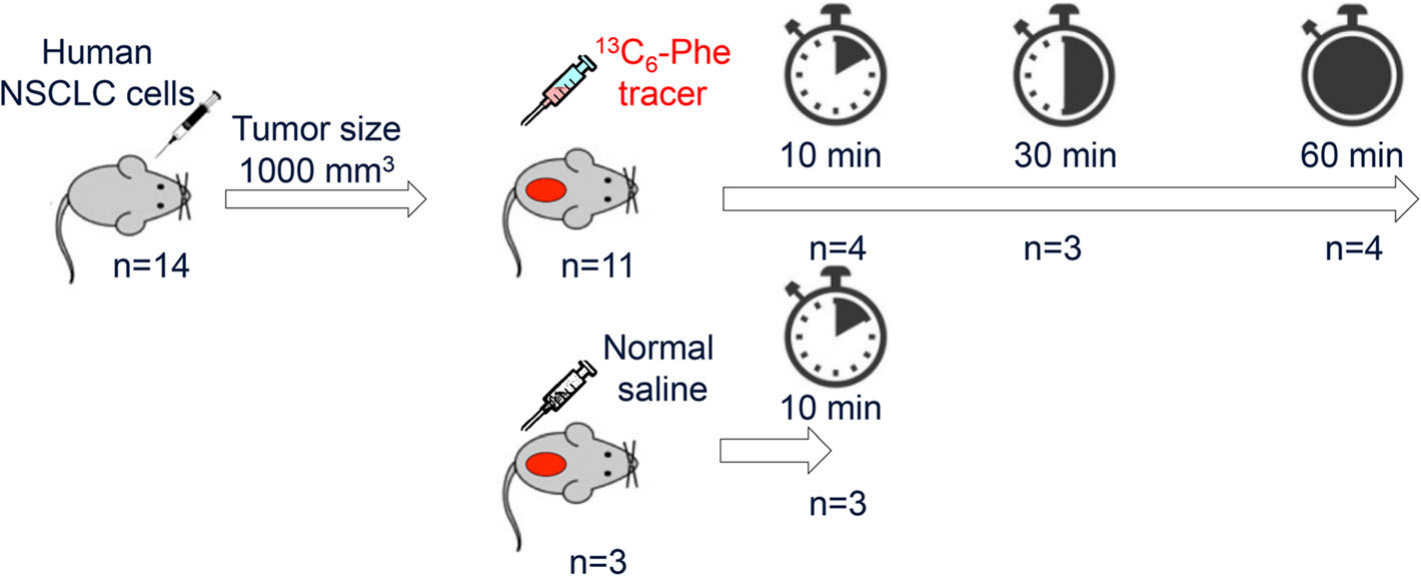
Eleven mice were injected with ring-^13^C_6_-Phe and three additional mice were injected with normal saline into the lateral tail vein when the tumors reached 1000 mm^3^. The tracer-infused mice were subsequently sacrificed at 10 min (*n* = 4), 30 min (*n* = 3), and 60 min (*n* = 4) after injection, and sham-treated mice were sacrificed after 10 min

We performed MALDI-FTICR-MSI analysis of mouse-xenograft lung tumor tissues 10 min (*n* = 4), 30 min (*n* = 3), and 60 min (*n* = 4) after tracer injection and after 10 min in a sham group (*n* = 3) to study ^13^C_6_-Phe and ^13^C_6_-Tyr kinetics (Fig. 1).

### Standard amino acids

**Fig. 2:**
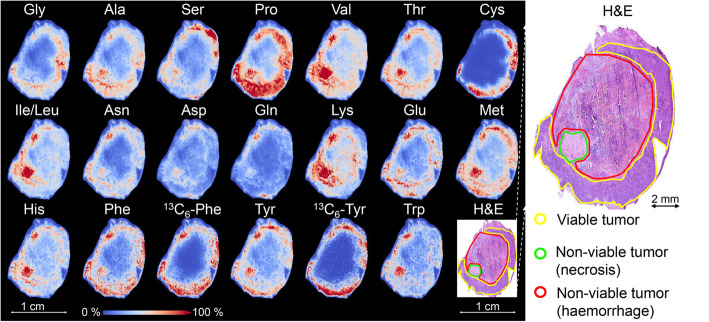
Distributions of 19 detected standard amino acids in a representative mouse-xenograft lung tumor tissue at 30 min after ^13^C_6_-Phe injection. All pixels were normalized to their root mean square value. The co-registered, hematoxylin and eosin stained (H&E) image shows the different histomorphological components of the tissue: viable tumor (yellow), non-viable tumor fraction (necrosis, green), and non-viable tumor region (hemorrhage, red)

Nineteen out of 20 standard amino acids were detected in all tumor sections and ^13^C_6_-Phe and ^13^C_6_-Tyr were exclusively detected in tracer-injected mice samples. MSI images of labeled and unlabeled amino acids in a representative tumor tissue at 30 min after ^13^C-Phe injection are shown in Fig. 2. All amino acids exhibit heterogeneous distributions, which correlate with the different morphological components of the tumor (mainly viable tumor and non-viable tumor). Most of unlabeled amino acids as well as ^13^C_6_-Phe and ^13^C_6_-Tyr showed similar spatial distributions with a higher abundance in viable tumor compared to the non-viable regions in the core of the tumor.

### ^13^C_6_-labeled Phe and Tyr

**Fig. 3:**
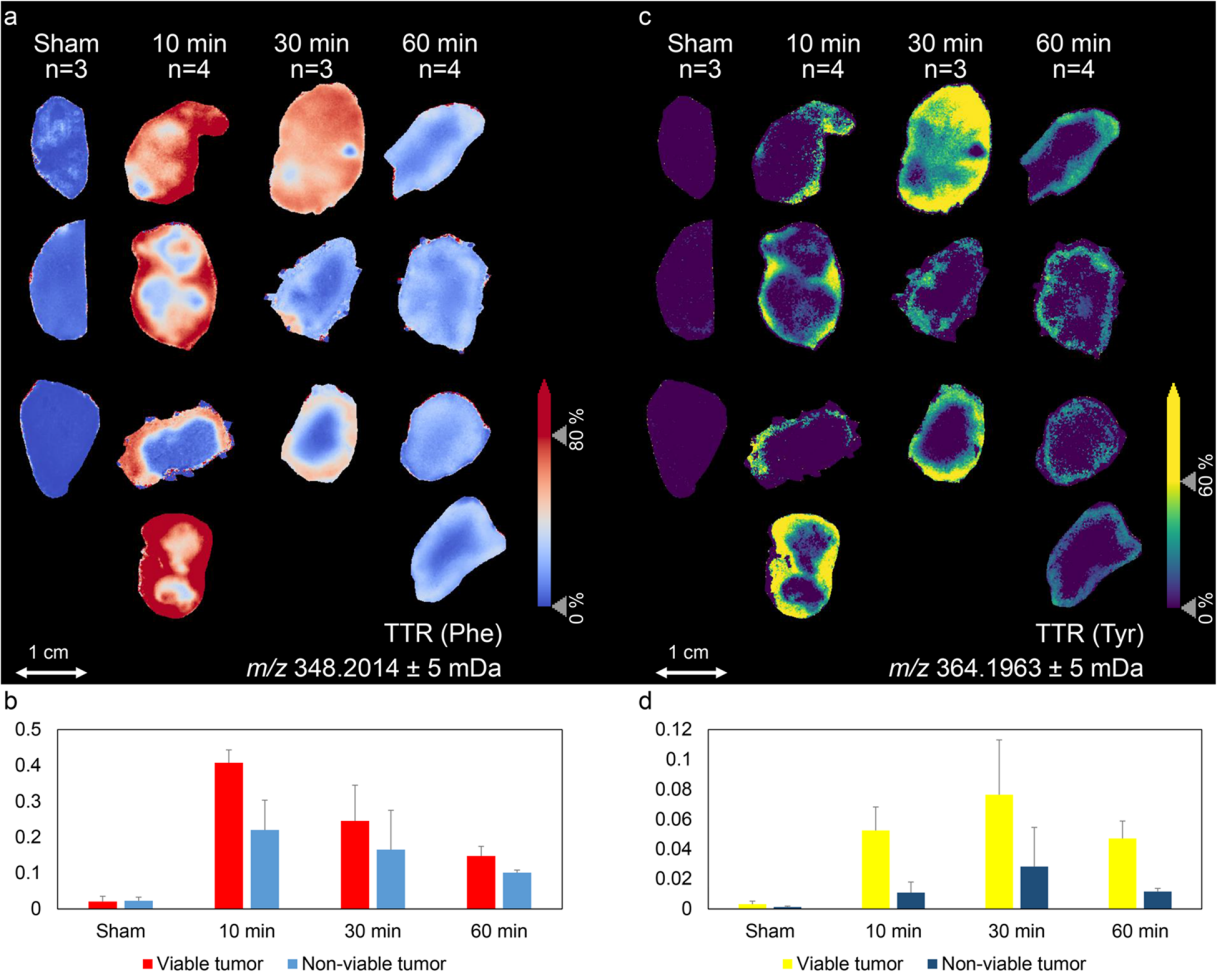
Distributions of ^13^C_6_-Phe (**a**) and ^13^C_6_-Tyr (**c**) in mouse-xenograft lung tumor tissues (n_total_ = 11) at 10, 30, and 60 min after tracer injection and at 10 min of control group (*n* = 3). Tracer-to-tracee (TTR) images for phenylalanine (**a**) and tyrosine (**c**) were calculated by normalizing the labeled amino acid signals to the intensities of their respective unlabeled versions. TTR values of Phe (**b**) and Tyr (**d**) were then differentiated annotated viable tumor and non-viable tumor regions for every time point

**Fig. 4:**
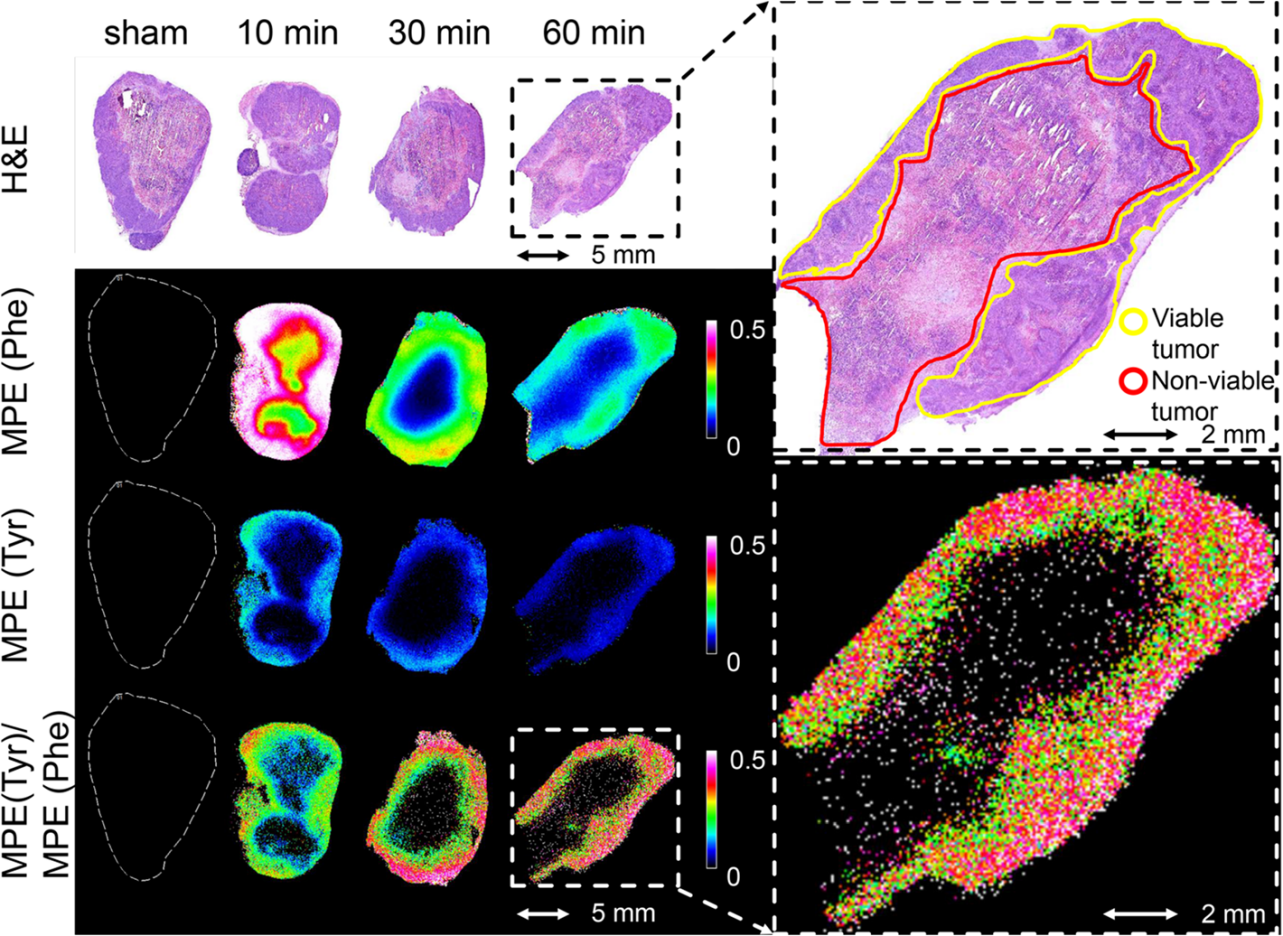
Visualizations of the molar percentage excess (MPE) for ^13^C_6_-Phe and ^13^C_6_-Tyr and the ratio of MPE (Tyr) to MPE (Phe) in representative tumor tissues at 10, 30, and 60 min after tracer injection and at 10 min of sham group. Their co-registered, hematoxylin and eosin stained (H&E) images are shown on the top. A magnification of the ratio of MPE (Tyr) to MPE (Phe) in one representative tissue section at 60 min and its co-registered H&E image is shown on the right

TTR and MPE values were used to assess the spatial enrichment of both ^13^C_6_-Phe and ^13^C_6_-Tyr in the tumor samples. The visualizations of TTR (Phe) and TTR (Tyr) across all tumor sections are shown in Fig. 3a and c, respectively. A differentiated quantitative analysis of TTR (Phe) and TTR (Tyr) in the annotated viable tumor and non-viable tumor fractions at every time point are shown in Fig. 3b and d, respectively. MPE (Phe), MPE (Tyr), ratio of MPE (Tyr) to MPE (Phe) in representative tumor tissues at 10, 30, and 60 min after tracer injection and at 10 min of control group are shown in Fig. 4. MPE values of Phe and Tyr in plasma samples from the same mice over the same time course were measured by GC-C-IRMS and are shown in Supplementary Figure 1.

The highest enrichment for ^13^C_6_-Phe was detected at 10 min after bolus injection followed by a decreasing trend over time. The overall enrichment of ^13^C_6_-Tyr was substantially lower than ^13^C_6_-Phe, and it had a delayed temporal trend compared with ^13^C_6_-Phe with its peak at 30 min. Additional experiments on plasma of the same mice show that both, ^13^C_6_-Phe and ^13^C_6_-Tyr, reached their highest level at 10 min after bolus injection followed by a decreasing trend over time (Supplementary Figure 1).

**Fig. 5:**
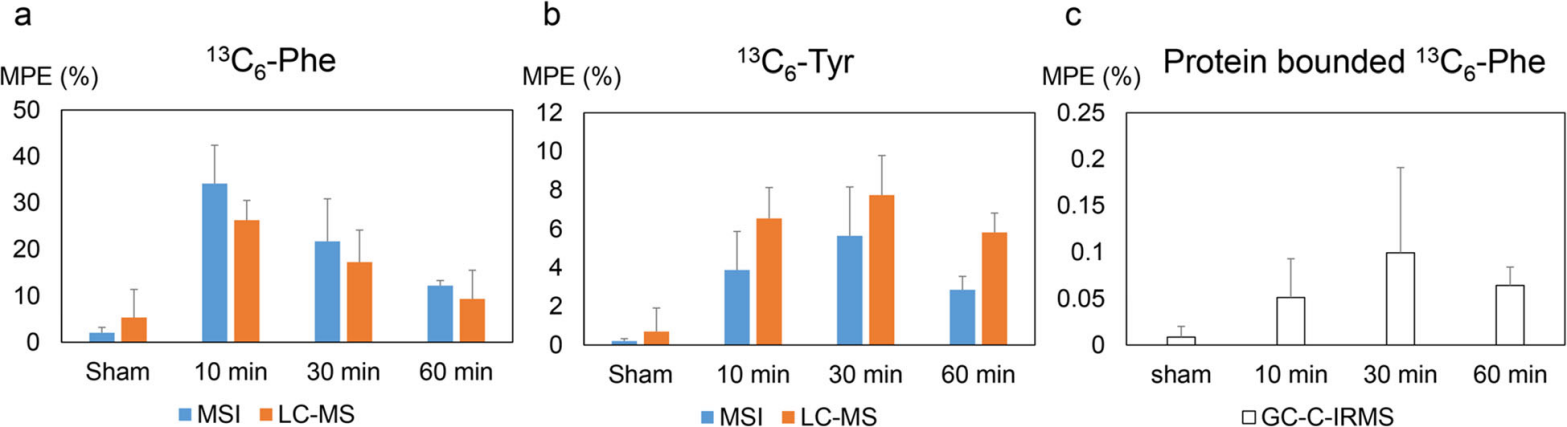
Overall enrichment of tissue free ^13^C_6_-Phe (**a**) and ^13^C_6_-Tyr (**b**) by MSI (blue) and LC-MS (orange), and ^13^C_6_-Phe protein enrichments (**c**) in homogenized tumor tissues by GC-C-IRMS. Enrichment is shown as mean MPE ± SE (%)

The MSI enrichments of ^13^C_6_-Phe and ^13^C_6_-Tyr were further complemented by LC-MS data acquired from homogenized tumor tissues, which showed a similar trend over time as the spatially convolved MSI data (Fig. 5a and b). Furthermore, the incorporation of ^13^C_6_-Phe into proteins in tumor tissue homogenates was investigated with GC-C-IRMS. The highest protein bounded ^13^C_6_-Phe was detected at 30 min and decreasing over time (Fig. 5c).

## Discussion

In this study, we used MALDI-FTICR mass spectrometry imaging (MSI) to study ^13^C_6_-Phe and ^13^C_6_-Tyr kinetics in mouse-xenograft lung tumor tissues at four different time points after injection. The use of MSI allowed us to detect all unlabeled and labeled amino acids simultaneously.

All observed amino acids exhibit heterogeneous distributions, which correlate with the different morphological components of the tumor (Fig. 2). For example, branched-chain amino acids (leucine, isoleucine, and valine) are most pronounced in the necrotic region, and all amino acids are much less abundant in the hemorrhagic region. This indicates the necessity to investigate amino acid kinetics in a spatially differentiated fashion using imaging technologies.

Looking at the labeled amino acids of interest, ^13^C_6_-Phe and ^13^C_6_-Tyr were found exclusively localized in the viable tumor region in contrast to their non-labeled equivalents, which are additionally present in necrotic regions.

When calculating the TTR and MPE values for both labeled amino acids for viable and non-viable tumor regions, the highest enrichments for ^13^C_6_-Phe and ^13^C_6_-Tyr were detected at 10 min and at 30 min after bolus injection, respectively, followed by a decreasing trend over time. Additional experiments on plasma of the same mice show that both, ^13^C_6_-Phe and ^13^C_6_-Tyr, reached their highest level at 10 min after bolus injection followed by a decreasing trend over time (Supplementary Figure 1). This together with the observation that the highest enrichment for ^13^C_6_-Tyr in the tumor was delayed indicates that ^13^C_6_-Tyr was subsequently transported to the tumor tissue after a Phe-to-Tyr conversion process, which is assumed to predominantly occur in the liver [13]. Moreover, viable and non-viable tumor fractions showed similar enrichment trends over time, but the viable tumor exhibited greater enrichment of both labeled amino acids than non-viable tumor at every time point (Fig. 3). This might be related to the higher metabolic activity of viable tumor cells and their higher perfusion over non-viable tumor tissue. Interestingly, the ratio of MPE (Tyr) to MPE (Phe), representing the Phe-to-Tyr turnover (in the tumor or elsewhere in the organism) was significantly higher in the outer rim of the viable tumor region (Fig. 4). This is a reflection of the intra-tumor heterogeneity within the viable tumor fraction, which might be attributed to spatial variation in perfusion, vascularization, cell growth, viability, or differences in metabolic activity [25].

These MSI enrichments of ^13^C_6_-Phe and ^13^C_6_-Tyr were further complemented by LC-MS and GC-C-IRMS data acquired from homogenized tumor tissues. The LC-MS data showed a similar trend over time as the spatially convolved MSI data, and thereby validated the accuracy of the MSI approach (Fig. 5a and b). Interestingly, the GC-C-IRMS data indicated a delayed incorporation of ^13^C_6_-Phe in the tumor protein synthesis as compared to the unbound labeled amino acids (Fig. 5), which might correlate with a lower clearance rate of ^13^C_6_-Phe in the tumor as compared to the liver where both ^13^C_6_-Phe and ^13^C_6_-Tyr were already cleared at 30 min and 60 min post-inoculation [17]. MALDI-MSI showed that clearance of both amino acids in the tumor was delayed compared to the liver, consequently delaying the incorporation of ^13^C_6_-Phe into the proteins synthetized in the tumor (Fig. 5c). Based on this, we can hypothesize that the incorporation of ^13^C_6_-Phe in the tumor protein synthesis presents a different kinetic compared to the liver progressive incorporation from 10 to 60 min. Nevertheless, while the liver tissue is fully viable and reasonably homogeneous, the heterogeneous tumor tissues were composed of both viable and non-viable parts. Only the viable tumor cells contribute to metabolic activities and so to protein synthesis in the tumor. Therefore, as the tumor tissue homogenates do not benefit from an estimation of the proportion of viable and non-viable parts, the protein incorporation results from homogenates should be interpreted with caution.

This again underlines the necessity to study kinetics in a spatially resolved manner. In that sense, this study demonstrates the usefulness of MSI to investigate spatial ^13^C_6_-Phe and ^13^C_6_-Tyr kinetics in tumor and also reflects inter-organ amino acid shifts. The translation to human samples will offer new insights in diagnostic molecular markers and tumor treatment.

## Conclusions

In this work, we demonstrated for the first time the spatiotemporal intra-tumoral distribution of the aromatic amino acid L-phenylalanine and its derivative L-tyrosine by MSI in tumor tissue. Furthermore, we showed the distribution of these molecular targets in relation to the tumor morphology, allowing us to monitor altered local amino acid metabolism in tumor cells and their microenvironment. Our approach can enhance our understanding on inter-organ amino acid metabolism and provide further insights to improve and develop novel strategies for cancer therapy.

## Supplementary Information

**Additional file 1: Supplementary Figure 1.** The molar percentage excess (MPE) for ^13^C_6_-Phe (left) and ^13^C_6_-Tyr (right) in plasma samples from the same mice over the same time course and measured by GC-C-IRMS.

## Data Availability

The data is available upon request from the corresponding author.

## Abbreviations

^13^C_6_-Phe: L-[ring-^13^C_6_]-phenylalanine
^13^C_6_-Tyr: L-[ring-^13^C_6_]-tyrosine
Phe: Phenylalanine
Tyr: Tyrosine
NSCLC: Non-small cell lung carcinoma
TTR: Tracer-to-tracee ratio
MPE: Molar percentage excess
MALDI: Matrix-assisted laser desorption/ionization
MSI: Mass spectrometry imaging
MALDI-FTICR-MSI: Matrix-assisted laser desorption/ionization Fourier-transform ion cyclotron resonance mass spectrometry imaging
MIMS: Multi-isotope imaging mass spectrometry
LC-MS: Liquid chromatography–mass spectrometry
GC-C-IRMS: Gas chromatography combustion isotope ratio mass spectrometry
TAHS: P-N,N,N-trimethylamonioanilyl N-hydroxysuccinimidylcarbamate iodide
DHB: 2,5-Dihydroxybenzoic acid
H&E: Hematoxylin and eosin staining
