# Supplementary material for: Mass spectrometry imaging of L-[ring-13C6]-labeled phenylalanine and tyrosine kinetics in non-small cell lung carcinoma
Source: Cancer Metab. 2021 Jun 11;9:26. doi: 10.1186/s40170-021-00262-9 (PMC8193875; doi:10.1186/s40170-021-00262-9)

**Mass spectrometry imaging of L-[ring-^13^C_6_]-labelled phenylalanine and tyrosine kinetics in non-small cell lung carcinoma**

Jianhua Cao, ^1^ Benjamin Balluff, ^1^ Martijn Arts, ^2^ Ludwig J. Dubois,^3^ Luc J. C. van Loon,^4^ Tilman M. Hackeng,^5^ Hans M. H. van Eijk, ^2^ Gert Eijkel,^1^ Lara R. Heij, ^2,6,7^ Zita Soons, ^2^ Steven W. M. Olde Damink, ^2,6^ Ron M.A. Heeren,^1*^

1 Maastricht MultiModal Molecular Imaging institute (M4I), Maastricht University, Maastricht, The Netherlands

2 Department of General Surgery (NUTRIM), Maastricht University, Maastricht, The Netherlands

3 The M-Lab, Department of Precision Medicine (GROW), Maastricht University, Maastricht, The Netherlands

4 Department of Human Biology (NUTRIM), Maastricht University, Maastricht, The Netherlands

5 Department of Biochemistry (CARIM), Maastricht University, Maastricht, The Netherlands

6 Department of General, Gastrointestinal, Hepatobiliary and Transplant Surgery, RWTH Aachen University Hospital, Aachen, Germany

7 Institute of Pathology, University Hospital RWTH Aachen, Aachen, Germany

**Supplementary Figure 1**. The molar percentage excess (MPE) for ^13^C_6_-Phe (left) and ^13^C_6_-Tyr (right) in plasma samples from the same mice over the same time course and measured by GC-C-IRMS.


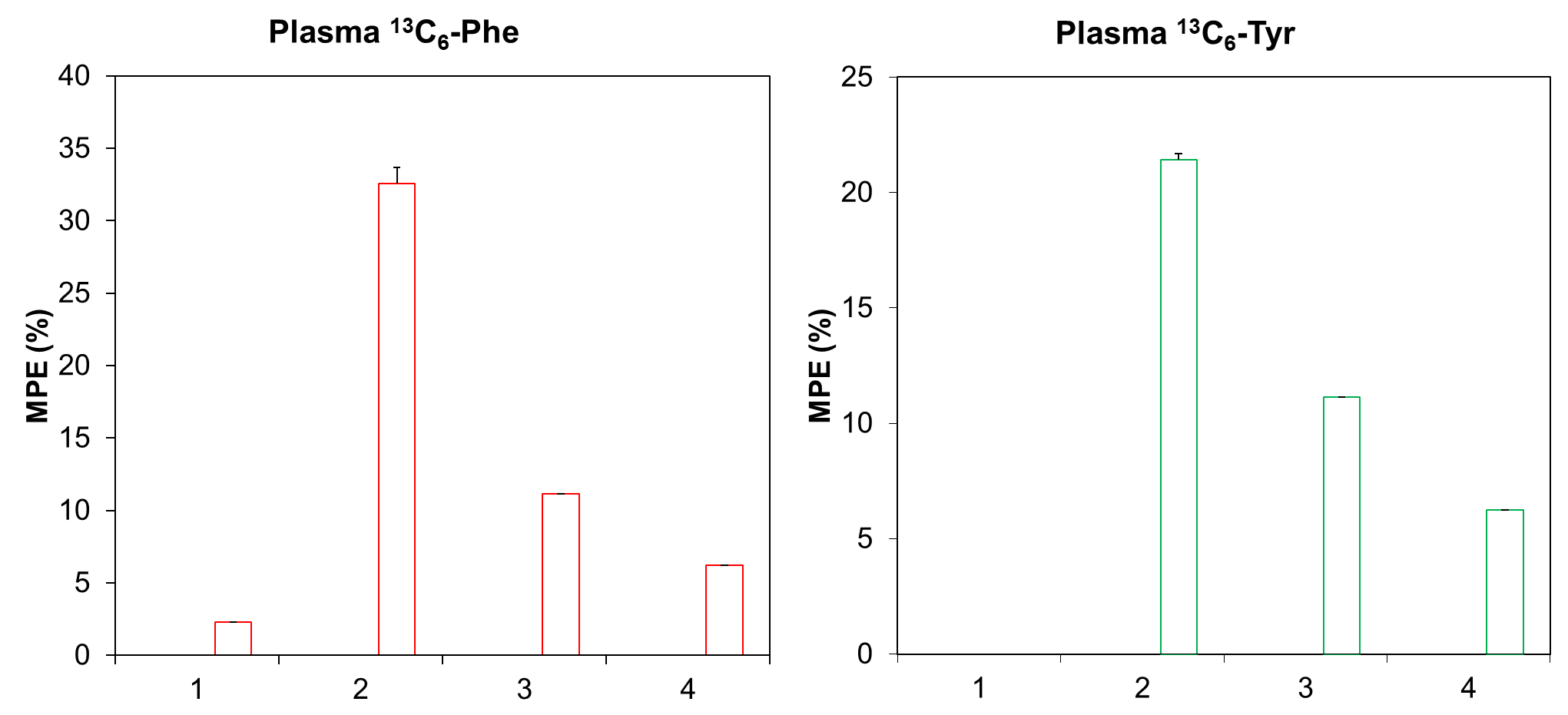

Supplement: Supplementary file 1 — Additional file 1: Supplementary Figure 1. The molar percentage excess (MPE) for 13C6-Phe (left) and 13C6-Tyr (right) in plasma samples from the same mice over the same time course and measured by GC-C-IRMS. [file 40170_2021_262_MOESM1_ESM.docx]
